# Adsorptive Removal of Heavy Metal Ions, Organic Dyes, and Pharmaceuticals by DNA–Chitosan Hydrogels

**DOI:** 10.3390/gels7030112

**Published:** 2021-08-06

**Authors:** Kayee Chan, Kohki Morikawa, Nobuyuki Shibata, Anatoly Zinchenko

**Affiliations:** 1 Graduate School of Environmental Studies, Nagoya University, Furo-cho, Chikusa-ku, Nagoya 464-8601, Japan; chan.kayee@a.mbox.nagoya-u.ac.jp (K.C.); vivi23.lvcafe@gmail.com (K.M.); 2 Nagoya Municipal Industrial Research Institute, 3-4-41, Rokuban, Atsuta, Nagoya 456-0058, Japan; shibata.nobuyuki@nmiri.city.nagoya.jp

**Keywords:** DNA, chitosan, hydrogel, adsorption, heavy metals, anionic dyes, cationic dyes, pharmaceuticals

## Abstract

DNA–chitosan (DNA–CS) hydrogel was prepared by in situ complexation between oppositely charged DNA and chitosan polyelectrolytes via electrostatic cross-linking to study its adsorption characteristics. The DNA–chitosan hydrogel matrix contains (i) cationic (NH_3_^+^) and anionic (PO_4_^−^) sites for electrostatic binding with ionic species, (ii) -OH and -NH_2_ groups and heteroaromatic DNA nucleobases for chelation of heavy metal ions, and (iii) DNA double-helix for recognition and binding to small organic molecules of various structures and polarities. DNA–CS hydrogels efficiently bind with Hg^2+^, Pb^2+^, Cd^2+^, and Cu^2+^ metal cations of significant environmental concern. Adsorption capacities of DNA–CS hydrogels for studied metal ions depend on hydrogel composition and pH of solution and reach ca. 50 mg/g at neutral pHs. Hydrogels with higher DNA contents show better adsorption characteristics and notably higher adsorption capacity to Hg^2+^ ions. Because of the co-existence of cationic and anionic macromolecules in the DNA–CS hydrogel, it demonstrates an affinity to both anionic (Congo Red) and cationic (Methylene Blue) dyes with moderate adsorption capacities of 12.6 mg/g and 29.0 mg/g, respectively. DNA–CS hydrogel can also be used for adsorptive removal of pharmaceuticals on conditions that their molecules are sufficiently hydrophobic and have ionogenic group(s). Facile preparation and multitarget adsorption characteristics of DNA–CS hydrogel coupled with sustainable and environmentally friendly characteristics render this system promising for environmental cleaning applications.

## 1. Introduction

Biomass-derived polymers are increasingly utilized in fields ranging from medicine and cosmetics to environmental engineering and construction. A broad variety of biomass polymers are available from agricultural, marine, and food wastes. For instance, chitosan and DNA can be obtained from waste products of the marine industry such as crab or shrimp shells (chitosan) or fish milt (DNA) [1,2]. Such biomass-derived polymers are particularly suitable for environmental applications thanks to their ecological safety. While virtually all known biomass polymers were utilized for construction of efficient absorbents for water and waste water cleaning applications [3,4], chitosan (CS) has drawn particular attention. CS contains plentiful amino and hydroxy groups and chitosan-based absorbents show superior adsorption characteristics toward transition metal ions [5,6], dyes [7], pharmaceuticals [8], and so on. Chitosan materials were also actively explored as drug delivery vehicles [9,10]. On the other hand, DNA materials were used for the removal of heavy metal ions [11,12,13], carcinogenic substances [14], dioxins [15], and organic molecules [16]. Recently, DNA hydrogels have gained marked consideration in environmental applications such as water treatment and analysis of specific pollutants [17].

Continuous appearance of new chemical classes of environmental pollutants during the past several decades has urged the development of not only efficient adsorbents for each class of pollutants, but also more complex and multitarget adsorbents capable of simultaneous sequestration of pollutants of different nature. Taking into account availability, environmental friendliness, and affinity of DNA and CS to different classes of environmental pollutants reported so far, here, we propose a unique type of DNA–CS composite hydrogel material and examine its adsorption capacities toward three classes of environmental pollutants.

Interaction of oppositely charged DNA and CS in aqueous solutions results in the formation of inter-polyelectrolyte complexes (IPECs) [18,19]. Application of such water-insoluble IPECs directly as adsorbents is limited by their poorly defined structure and non-reversible aggregative behaviour. To overcome these limitations, recently, we designed and succeeded in the preparation of interpolyelectrolyte hydrogel material from DNA and CS with a well-defined nano- and microstructure [20]. In contrast to a vast number of hybrid polymeric adsorbents that are usually prepared by a covalent cross-linking of two or more polymer components, stable DNA–CS hydrogels can be formed as a result of electrostatic interactions between oppositely charged macromolecules and require no additional cross-linking by reactive chemicals [20,21].

IPECs from natural and synthetic polyelectrolytes, including those of DNA and chitosan, have been studied intensively [18,19,20,22,23,24] and applied for the removal of environmental pollutants such as sulphates [25,26], phosphates and nitrates [27], heavy metal ions [28], bisphenol A [29], nanomaterials [30], and so on. However, the adsorption properties of IPEC hydrogels have not been fully explored and the idea of combining polyions as active adsorption components into a single hydrogel matrix has not been fully addressed in previous studies. The present report shows a proof-of-concept design of multitarget DNA–CS hydrogel adsorbent and its application for the removal of (i) heavy metal ions, (ii) cationic and anionic organic dyes, and (iii) pharmaceuticals.

## 2. Results and Discussion

### 2.1. Preparation of DNA–CS Hydrogel Adsorbent

**Figure 1:**
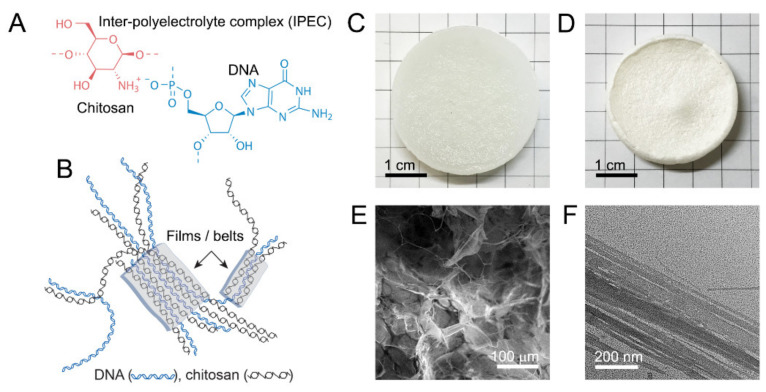
DNA–CS hydrogel formation and structure. (**A**) Formation of an ionic bond between chitosan and DNA monomer units. (**B**) Schematic representation of DNA and chitosan electrostatic interaction and formation of two-dimensional domains of belts or films. (**C**) Photographic image of DNA–CS hydrogel after washing. (**D**) Photographic image of freeze-dried DNA–CS hydrogel. (**E**) Typical SEM image of freeze-dried DNA–CS hydrogel. (**F**) Typical TEM image of DNA–CS films and fibrils in DNA–CS hydrogel.

DNA–chitosan hydrogels were prepared in a similar manner as described recently [20]. In brief, dispersion of chitosan powder in 1% DNA solution was gradually acidified using glucono-D-lactone (GDL), whose hydrolysis resulted in the pH change from neutral to slightly acidic (pH 5.0) and consequent dissolution of chitosan. Electrostatic interaction of the dissolving chitosan with oppositely-charged DNA (Figure 1A) resulted in the formation of a polyelectrolyte network of DNA–CS hydrogel (Figure 1B,C).

Swelling behavior as well as stability and mechanical characteristics of DNA–CS hydrogels and their dependences on a ratio of cationic and anionic components were discussed in detail elsewhere [20]. An SEM image of freeze-dried DNA–CS hydrogel (Figure 1D) shows that the hydrogel scaffold is composed of films and microfibrils (Figure 1E). A TEM image of freeze-dried hydrogel fragments (Figure 1E) suggests that the films observed by SEM are formed by lateral association of oppositely-charged DNA and chitosan macromolecules into two-dimensional planar structures of various widths by a mechanism proposed in Figure 1B. DNA–CS hydrogels prepared by the above method possess sufficient mechanical stability in aqueous solutions [20] and can be applied for the adsorptive removal of heavy metal ions, cationic and anionic dyes, and organic molecules of mass-produced pharmaceuticals. Hydrogels with different NH_2_/PO_3_ ratios were prepared, and they are abbreviated hereafter as CS60 (NH_2_/PO_3_ = 0.70), CS90 (NH_2_/PO_3_ = 0.98), and CS120 (NH_2_/PO_3_ = 1.29), whereas most adsorption experiments were carried out using the stoichiometric CS90 hydrogel.

### 2.2. Adsorption of Heavy Metal Ions by DNA–CS Hydrogel

Both DNA and chitosan are well known by their high affinity to a broad range of metal cations [5,31]. Chitosan interacts with metal cations predominantly by chelating of metal ions with amines [5]. The mechanism of DNA interaction with metal cations varies depending on the nature of metal cations. This can be either purely electrostatic, i.e., interaction of negatively charged DNA phosphates with metal cations, or can involve coordination of metal ions with nucleobases [31,32]. The latter mechanism dominates in DNA binding with transition metal ions and is characterized by higher binding constants [31].

Adsorption experiments were performed by placing a small piece of DNA–CS hydrogel into a solution of metal ions. To address multitarget adsorption characteristics of DNA–CS hydrogel and to gain insight into the competition of metal ions for binding to DNA–CS adsorbent, a solution for adsorption experiment was prepared by mixing 50 ppm solutions of four metal ions of environmental concern: Hg^2+^, Pb^2+^, Cd^2+^, and Cu^2+^. Concentrations of metal ions during adsorption were measured by ICP-AES spectroscopy.

**Figure 2:**
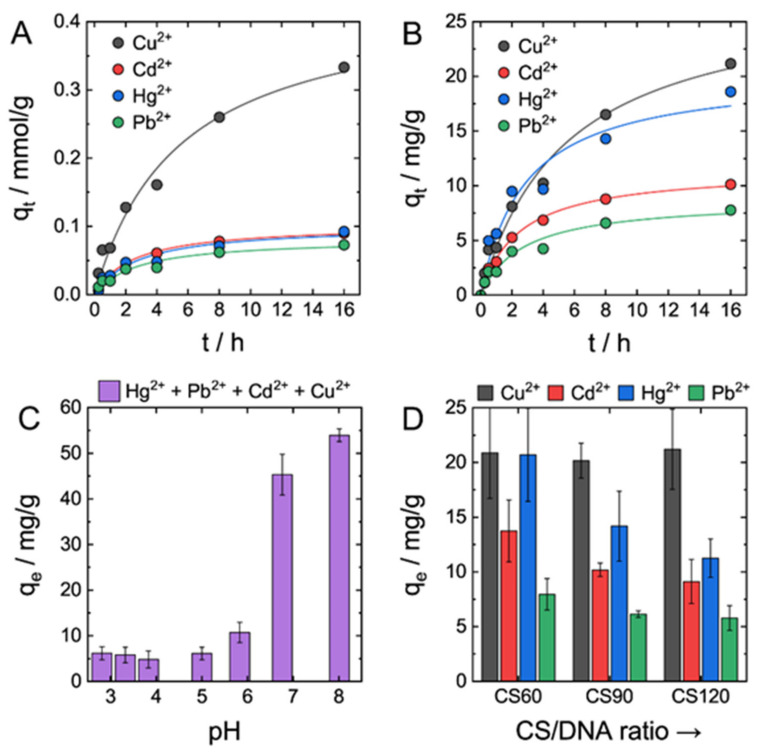
Adsorption of metal ions by DNA–CS hydrogel. (**A**,**B**). Kinetics curves of Cd^2+^, Cu^2+^, Hg^2+^, and Pb^2+^ adsorption by DNA–CS hydrogel from a solution containing 50 ppm of each ion expressed in mmol/g (**A**) and mg/g (**B**) units. Lines are fitting curves of the pseudo-second-order kinetic model. (**C**) Dependence of DNA–CS hydrogel adsorption capacities for metal ions on solution pH. (**D**) Adsorption capacities of DNA–CS hydrogels with different NH_2_/PO_3_ ratios (CS60—0.70, CS90—0.98, and CS120—1.29) for metal ions. Error bars in Figures C and D are standard deviations from average of duplicate independent experiments.

Time-dependent adsorption data (Figure 2A,B) show steady, but relatively slow adsorption of all four types of ions by DNA–CS hydrogel. Adsorption data of each individual metal ion and the total amount of ions were best fit by the pseudo-second-order kinetic model compared with the pseudo-first-order kinetic model. DNA–CS hydrogel had clear, ca. threefold in terms of adsorption capacity, preference to Cu^2+^ ions compared with Hg^2+^, Pb^2+^, and Cd^2+^ (Figure 2A), which were absorbed at similar mmol/g quantities. Weight-based adsorption capacities of DNA–CS hydrogel for metal ions decreased in the order Cu^2+^ > Hg^2+^ > Pb^2+^ > Cd^2+^ (Figure 2B). The total amount of absorbed metal ions was ca. 60 mg/g or 0.6 mmol/g, which is somewhat low compared with, for instance, pure chitosan absorbents with typical absorption capacities of 1–2 mmol/g [5,33,34]. The reason for lower adsorption capacity of the DNA–CS adsorbent is apparently owing to its structure (Figure 1B). Indeed, DNA–CS adsorbent is formed by the ionic cross-linking between protonated NH_2_ groups of CS and phosphate groups of DNA; therefore, part of the NH_2_ groups of CS taking part in NH_3_^+^PO_4_^−^ bonds’ formation cannot chelate metal ions.

The affinity of chitosan to metal ions was studied by many research groups using different techniques. Stronger affinity of chitosan to Cu^2+^ and Hg^2+^ ions was reported compared with Cd^2+^ and Pb^2+^, yet there was no general agreement on relative affinity to metal ions in each of these two pairs [35], likely because of differences in pH and other experimental conditions. However, in a majority of studies, the strongest affinity of chitosan was ascribed to Cu^2+^ ion [35]. DNA forms a strong complex with Hg^2+^ ion, which binds exclusively to DNA bases and is strongly held between two DNA helices [36]. Three other metal ions interact with DNA by either electrostatic binding with phosphates or coordination with nucleobases and DNA affinity to these ions changes as Cu^2+^ > Pb^2+^ > Cd^2+^ [32]. Cu^2+^ has a higher binding constant than Pb^2+^ and Cd^2+^ ions owing to its higher affinity to DNA bases [37]. Taking into account adsorption properties of DNA and chitosan, the preference of DNA–CS hydrogel to Cu^2+^ was not surprising. Unexpectedly low adsorption capacity of DNA–CS hydrogel to Hg^2+^ is explained by its competition with Cu^2+^ ion for DNA intercalation [38].

The adsorbed amount of metal ions depended strongly on solution pH (Figure 2C). Adsorption capacities of metal ions in acidic solution at pHs 2–5 did not vary much and were low (ca. 5–7 mg/g). At pH ≥ 7, adsorption capacities increased drastically by ca. 10-fold compared with the acidic pHs. pH-sensitive adsorption is in good agreement with earlier studies [33] and was expected based on mechanisms of metal ions binding to chitosan and DNA. Transition metal ions coordinate preferably with amino groups of CS and nitrogen atoms of DNA nucleobases. Protonation of these binding sites at low pHs prevents coordination of studied ions with DNA–CS adsorbent and additionally causes electrostatic repulsion between metal dictation and adsorption site.

To address individual contributions of DNA and chitosan to the adsorption performance of DNA–CS hydrogel, three types of hydrogels with different DNA/chitosan ratios were tested (CS60, CS90, and CS120). CS90 represents nearly stoichiometric IPEC with the same amount of cationic and anionic groups, whereas CS60 and CS120 are enriched by either DNA or CS, respectively [20]. Figure 2D shows that an increase in DNA fraction in the hydrogel adsorbent resulted in the increase of the total absorbed amount of metal ions. No changes were measured in the Cu^2+^ adsorption capacity of all three adsorbents, but the adsorption capacities of three other metal ions increased markedly by 1.5–2-fold from CS120 containing less DNA to CS60 having higher DNA contents. Higher adsorption capacities of CS60 hydrogel toward Hg^2+^ are ascribed to a very strong binding constant of DNA to Hg^2+^ [36]. Based on the past literature, affinity of DNA to Cd^2+^ and Pb^2+^ are considered to be comparable to that of chitosan; therefore, an increase of their adsorption capacities by DNA-rich hydrogel CS60 was ascribed to the presence of a larger number of available phosphate groups in the hydrogel, favoring stronger electrostatic binding to cationic metal ions.

### 2.3. Adsorption of Anionic and Cationic Dyes and Pharmaceuticals by DNA-CS Hydrogel

**Figure 3:**
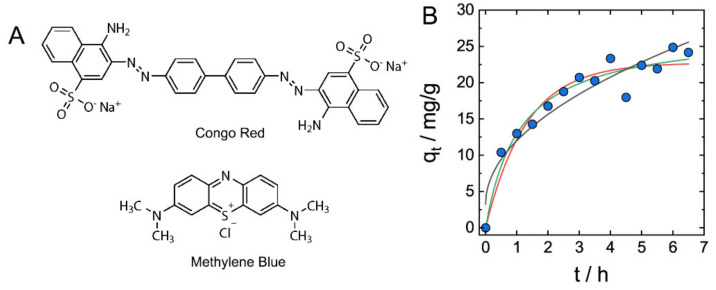
Adsorption of cationic and anionic dyes by DNA–CS hydrogels. (**A**). Chemical structure of Congo Red and Methylene Blue. (**B**). Kinetics of Methylene Blue absorption by DNA–CS hydrogel. Lines are fitting to pseudo-first-order (red), pseudo-second-order (green), and interparticle diffusion (black) kinetics models.

In contrast to typical hydrogel adsorbents that are either anionic or cationic polymeric networks, co-existence of anionic and cationic sites in a single DNA–CS material suggests a possibility of the adsorption of both anionic and cationic species by electrostatic mechanism. Batch adsorption experiments using typical anionic and cationic dyes, Congo Red and Methylene Blue (Figure 3A), were performed by soaking DNA–CS hydrogels (CS90) in solutions of either dye of 50 mg/L concentration, and adsorption processes were monitored spectroscopically (Supplementary Figure S1).

Adsorption kinetics data of Methylene Blue adsorption by DNA–CS hydrogel are shown in Figure 3B. The adsorption of MB dye is characterized by relatively slow kinetics that requires hours for establishing the adsorption equilibrium, yet it is faster than the adsorption of metal ions (Figure 2A,B). The kinetics data were fit using pseudo-first-order, pseudo-second-order, and interparticle diffusion kinetic models, and the best fit was found for the pseudo-second-order kinetic model (R = 0.9773), similarly to the adsorption of metal ions.

Both anionic and cationic dyes were adsorbed by DNA–CS hydrogels, with adsorption capacities shown in Table 1. Considering purely electrostatic mechanism of dyes’ adsorption by hydrogels, only ca. 5% of charged groups of DNA and CS were involved in binding with dye molecules. Apart from weak electrostatic interaction of polyions with monovalent ions of dyes, low adsorption capacities are related to the structure of DNA–CS hydrogel discussed above. As long as a significant part of cationic and anionic groups of DNA and chitosan is consumed for ionic bonding during IPEC formation, they do not take an active part in the electrostatic adsorption of dyes. The adsorption capacity of DNA–CS hydrogel for the cationic MB dye was about 2.5 times higher than for the anionic CR dye. This difference is furthermore remarkable when adsorption capacities are compared in mmol/g units (Table 2). We suggest that more efficient binding of MB to DNA–CS complex is favored by intercalation of MB into DNA double-helix and formation of stable complex [39], in contrast to the predominately electrostatic interaction of CR with chitosan.

**Figure 4:**
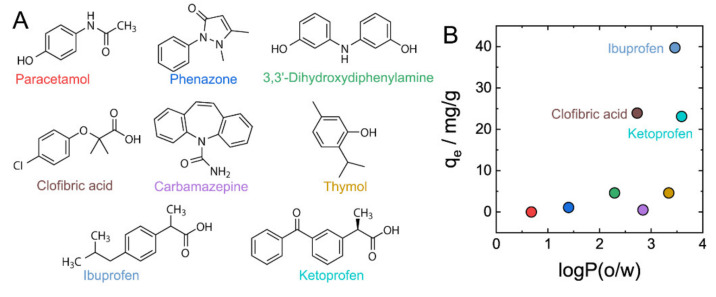
Adsorption of eight representative pharmaceuticals by DNA–CS hydrogels. (**A**). Chemical structures of studied pharmaceuticals. (**B**). Correlation between adsorption capacities of DNA–CS hydrogels for pharmaceuticals and their octanol/water partition coefficients.

To gain further insight into the relationship between the structure of absorbing molecules and adsorption by DNA–CS hydrogels, and taking into account the practical importance of adsorptive removal of mass-produced pharmaceuticals in water treatment [40], we selected eight water-soluble mass-produced pharmaceuticals (Figure 4A) and studied their adsorption by DNA–CS hydrogels. Concentrations of pharmaceuticals before and after adsorption were measured by UV/vis spectroscopy (Supplementary Figure S1).

The adsorption capacity was strongly affected by the chemical structure of absorbing molecules (Figure 4B and Table 2) and varied from nearly 0 mg/g to 40 mg/g. To look into the correlation between the polarity of pharmaceutical molecules and their uptake by DNA–CS hydrogels, 1-octanol/water solvent partitioning coefficients (logP_o/w_) were calculated (Table 2) to plot against adsorption capacities in Figure 4B. Figure 4B shows that there was no clear correlation between logP_o/w_ and adsorption capacities; however, only hydrophobic molecules with logP_o/w_ >2.5 were adsorbed at significant quantities. A closer look at the chemical structure of pharmaceuticals with higher uptake ratios revealed that all of them (ibuprofen, clofibric acid, and ketoprofen) possessed an ionogenic carboxylic group. This structural feature points to the importance of electrostatic interaction of pharmaceutical molecules and DNA–CS hydrogel, and suggests a scenario of electrostatic binding of anionic pharmaceutical molecules to cationic chitosan that is additionally stabilized by hydrophobic interactions with DNA–CS complex.

## 3. Conclusions

We demonstrated that DNA–CS interpolyelectrolyte hydrogel adsorbent prepared by gradual charging of CS in concentrated solutions of DNA can be used for adsorptive removal of a broad range of environmental pollutants including heavy metal ions, cationic and anionic dyes, and mass-produced pharmaceuticals molecules, owing to the integration of various binding sites into a single hydrogel adsorbent. Adsorptions of every type of pollutants were characterized by somewhat slow kinetics and were best fit by the pseudo-second-order kinetic model. The slow kinetics is presumably a result of structural transformations inside IPEC accompanying binding of adsorbing chemical to DNA–CS scaffolds. IPEC hydrogels have ca. 50 mg/g capacity towards heavy metal ions, with a particular preference to Cu^2+^ and Hg^2+^. Owing to the coexistence of anionic and cationic macromolecules in DNA-CS, both cationic and anionic dyes are adsorbed with capacities of several tens of mg/g. Adsorption experiments using pharmaceuticals made clear that higher hydrophobicity of an absorbing molecule and the presence of an ionogenic group favor adsorption of organic molecules by DNA–CS hydrogel with 20–40 mg/g adsorption capacities. Proposed in this study, multitarget DNA–CS adsorbent prepared from biomass polymers represents a sustainable and renewable composite material that is environmentally benign and “green” by design.

## 4. Materials and Methods

### 4.1. Materials

DNA sodium salt (>5 kbp, ca. 90% purity), CS (50 kDa–190 kDa, 75–85% deacetylation), and D-(+)-glucono-*δ*-lactone (GDL) were purchased from Sigma-Aldrich (St. Louis, MI, USA). Sodium hydroxide (NaOH), 6 M solution of hydrochloric acid, and sodium chloride (NaCl) were used as received. Congo Red from Kishida Chemical Co., Ltd. (Osaka, Japan) and 1% Methylene Blue solution (MB) form Kanto Chemical Co., Inc. (Tokyo, Japan) were used for dye adsorption experiments. Carbamazepine, ibuprofen, and ketoprofen were purchased from Wako Pure Chemical Industries, Ltd. (Osaka, Japan); clofibric acid, paracetamol, thymol, and 3,3’-Dihydroxydiphenylamine were purchased from Tokyo Chemical Industry Co., Ltd. (Tokyo, Japan); and phenazone was purchased from Sigma-Aldrich (Japan). Standard solutions of 100 ppm (JCSS) of Cd(NO_3_)_2_, Cu(NO_3_)_2_, HgCl_2_, and Pb(NO_3_)_2_ in 0.1 M HNO_3_ were purchased from FUJIFILM Wako Pure Chemical Corporation (Osaka, Japan). Milli-Q water was purified by Purelab Chorus 1 Life Science apparatus and used in all experiments.

### 4.2. Methods

UV/Vis spectroscopy. UV/vis spectra of aqueous solutions of dyes or pharmaceuticals were recorded on a Jasco V-630 spectrophotometer (Japan) in 1 mL quartz cells with an optical path 1 cm at 25 °C.

Inductively coupled plasma atomic emission spectroscopy (ICP-AES). Concentration of metal ions in solutions was determined by an inductively coupled plasma atomic emission spectrometer (ICP-AES, SEIKO SPS3520, Tokyo, Japan) after an appropriate dilution.

Scanning Electron Microscopy (SEM). SEM observations of freeze-dried (Eyela FDU-1200, Tokyo, Japan) hydrogels were performed at room temperature on a JSM-6610 microscope (JEOL, Tokyo, Japan) at an acceleration voltage of 15 kV.

Transmission electron microscopy (TEM). TEM observations were performed on a JEM-2100 Plus microscope (JEOL, Japan) at 200 kV acceleration voltage at room temperature. Samples of DNA–CS hydrogels were prepared by grinding wet hydrogels with a mortar and then diluting with Milli-Q water (ca. 10-fold by weight). Then, 50 μL of dispersion was placed on a piece of parafilm and covered by a carbon-coated copper grid (Alliance Biosystems, Osaka, Japan). The droplet was removed with a filter paper after 3 min, and the copper grid was placed on a droplet of Uranyless staining solution (Electron Microscopy Sciences, Hatfield, PA, USA) for another 3 min. Finally, the droplet of Uranyless solution was soaked by a filter paper and the grid was dried in a dry box at relative humidity <10% overnight.

Calculations of octanol–water partition coefficients of organic chemicals. The web-based tool Molinspiration (http://www.molinspiration.com/, accessed on 1 February 2021) was used to calculate octanol–water partition coefficients (logP_o/w_) of pharmaceutical molecules.

### 4.3. Samples’ Preparation

Preparation of DNA–CS hydrogels. DNA stock solution (1.0% *w*/*v*) was prepared by stirring of DNA in Milli-Q water overnight. Typically, a chitosan powder of 60, 90, or 120 mg was first added into 3 mL of DNA solution at vigorous 500 rpm stirring, and then 70 mg of glucono-*δ*-lactone (GDL) powder was admixed into the chitosan dispersion under continuous stirring for 10 min. After an additional 30 min, the stirring was stopped and the gelated mixture was kept opened overnight in the air. The hydrogel was washed twice in 500 mL by Milli-Q water and stored in Milli-Q water under ambient conditions. DNA–CS aerogels were prepared using FDU-1200 lyophilizer (Eyela, Japan).

Adsorption of heavy metal ions by DNA–CS hydrogel. Solutions of metal ions were prepared by dilution of their 100 ppm standard solutions (JCSS) with Milli-Q water and Tris-HCl buffer at different ratios to adjust pH between 2 and 8. Pieces of DNA–CS hydrogels (ca. 50–100 mg) were placed into 5 mL solutions containing 50 ppm of Hg^2+^, Pb^2+^, Cd^2+^, and Cu^2+^, respectively, and changes in their concentrations were measured by ICP-AES.

Adsorption of anionic and cationic dyes by DNA–CS hydrogel. Pieces of DNA–CS hydrogels (ca. 0.5 g) were placed into 12 mL solutions containing 50 mg/L of dyes for 2 days and changes in their concentrations were measured by UV/vis spectroscopy at 498 nm for Congo Red and 663 nm for Methylene Blue.

Adsorption of pharmaceuticals by DNA–chitosan hydrogel. Pieces of DNA–CS hydrogels (ca. 0.8–1 g) were placed into 4 mL solutions containing pharmaceuticals of 1 mM concentration for 2 days and changes in their concentrations were measured by UV/vis spectroscopy at wavelengths of maximum absorbance: carbamazepine—285 nm, ibuprofen—222 nm, clofibric acid—227 nm, paracetamol—243 nm, phenazone—242 nm, ketoprofen—260 nm, thymol—274 nm, and 3,3’-dihydroxydiphenylamine—278 nm.

## Figures and Tables

**Table 1:** Adsorption capacities of DNA–CS hydrogel (CS90) for CR and MB dyes.

Dye	q_e_, mg/g	q_e_,^1^ mmol/g
Congo Red (CR, anionic)	12.6	0.014
Methylene blue (MB, cationic)	29.0	0.102

^1^ Counterions of the dyes (Cl^−^ and Na^+^) were not included.

**Table 2:** 1-Octanol/water solvent partitioning coefficients (logP_o/w_) and adsorption capacities of DNA–CS hydrogels for pharmaceuticals.

Pharmaceuticals	logP_o/w_	Adsorption Capacity, mg/g
Paracetamol	0.68	<0.1
Phenazone	1.40	1.1
3,3’-Dihydroxydiphenylamine	2.29	4.6
Clofibric acid	2.73	23.9
Carbamazepine	2.84	0.5
Thymol	3.34	4.6
Ibuprofen	3.46	39.7
Ketoprofen	3.59	23.1

## Supplementary Materials

The following are available online at https://www.mdpi.com/article/10.3390/gels7030112/s1, Figure S1: UV/vis absorbance spectra of dyes (A) and pharmaceuticals (B) before and after adsorption by DNA–CS hydrogels.
