# Supplementary material for: Adsorptive Removal of Heavy Metal Ions, Organic Dyes, and Pharmaceuticals by DNA–Chitosan Hydrogels
Source: Gels. 2021 Aug 6;7(3):112. doi: 10.3390/gels7030112 (PMC8395854; doi:10.3390/gels7030112)
Supplement: Supplementary file 1 [file gels-07-00112-s001.zip › gels-1264418-supplementary.pdf]

Supplementary Material

Adsorptive Removal of Heavy Metal Ions, Organic Dyes, and Pharmaceuticals by DNA–Chitosan Hydrogels

Kayee Chan [1,†], Kohki Morikawa [1,†], Nobuyuki Shibata [2], and Anatoly Zinchenko [1,*]

[1] Graduate School of Environmental Studies, Nagoya University, Furo-cho, Chikusa-ku, Nagoya 464-8601, Japan; chan.kayee@a.mbox.nagoya-u.ac.jp (K.C.); vivi23.lvcafe@gmail.com (K.M.)

[2] Nagoya Municipal Industrial Research Institute, 3-4-41, Rokuban, Atsuta, Nagoya 456-0058, Japan; shibata.nobuyuki@nmiri.city.nagoya.jp

* Correspondence: zinchenko@urban.env.nagoya-u.ac.jp; Tel.: +81-52-789-4771

† These authors contributed equally to this work.

1. UV-vis absorbance spectra.

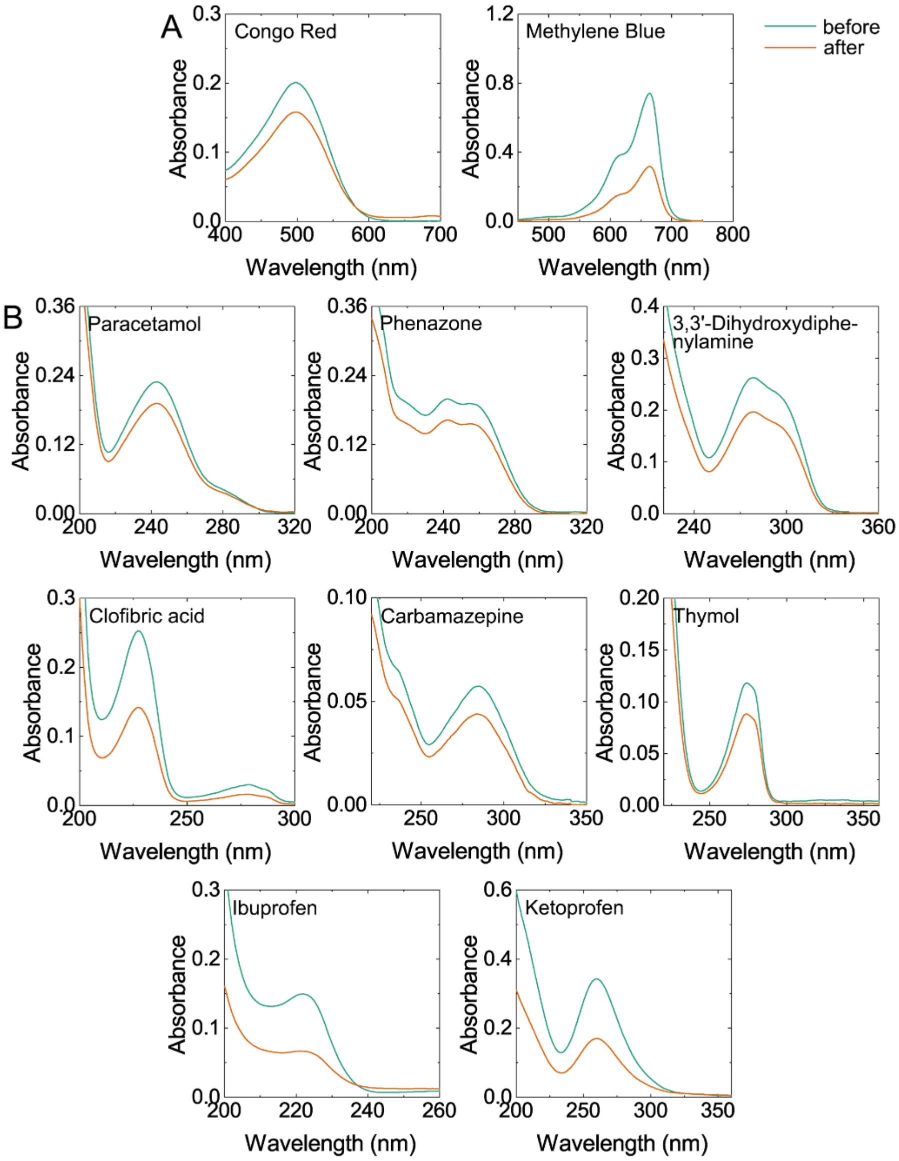

Figure S1. UV-vis absorbance spectra of industrial dyes (**A**) and pharmaceuticals (**B**) before and after 48 hours of absorption by DNA-CS hydrogel used to calculate their absorption capacities. The experimental conditions are given in the Experimental Section.
